# Supplementary material for: New digital anatomical data of Keichousaurus hui (Reptilia: Sauropterygia) and its phylogenetic implication
Source: PeerJ. 2025 Mar 31;13:e19012. doi: 10.7717/peerj.19012 (PMC11967422; doi:10.7717/peerj.19012)
Supplement: Table S1 [file peerj-13-19012-s001.docx]

| 0000100000 | 1020021000 | 1101**1**01000 | 002030011**0** | 0302100011 |
| --- | --- | --- | --- | --- |
| 001?0**1**2011 | ?10000200? | 0100001100 | 000**{0 1}1**0001**2** | 1100011010 |
| 1100000110 | 1100110010 | 0200011111 | 1111010211 | 0011111111 |
| 1000210111 | 1112110110 | 0110001013 | 0 |  |
