# Supplementary figures and images for: New digital anatomical data of Keichousaurus hui (Reptilia: Sauropterygia) and its phylogenetic implication
Source: PeerJ. 2025 Mar 31;13:e19012. doi: 10.7717/peerj.19012 (PMC11967422; doi:10.7717/peerj.19012)

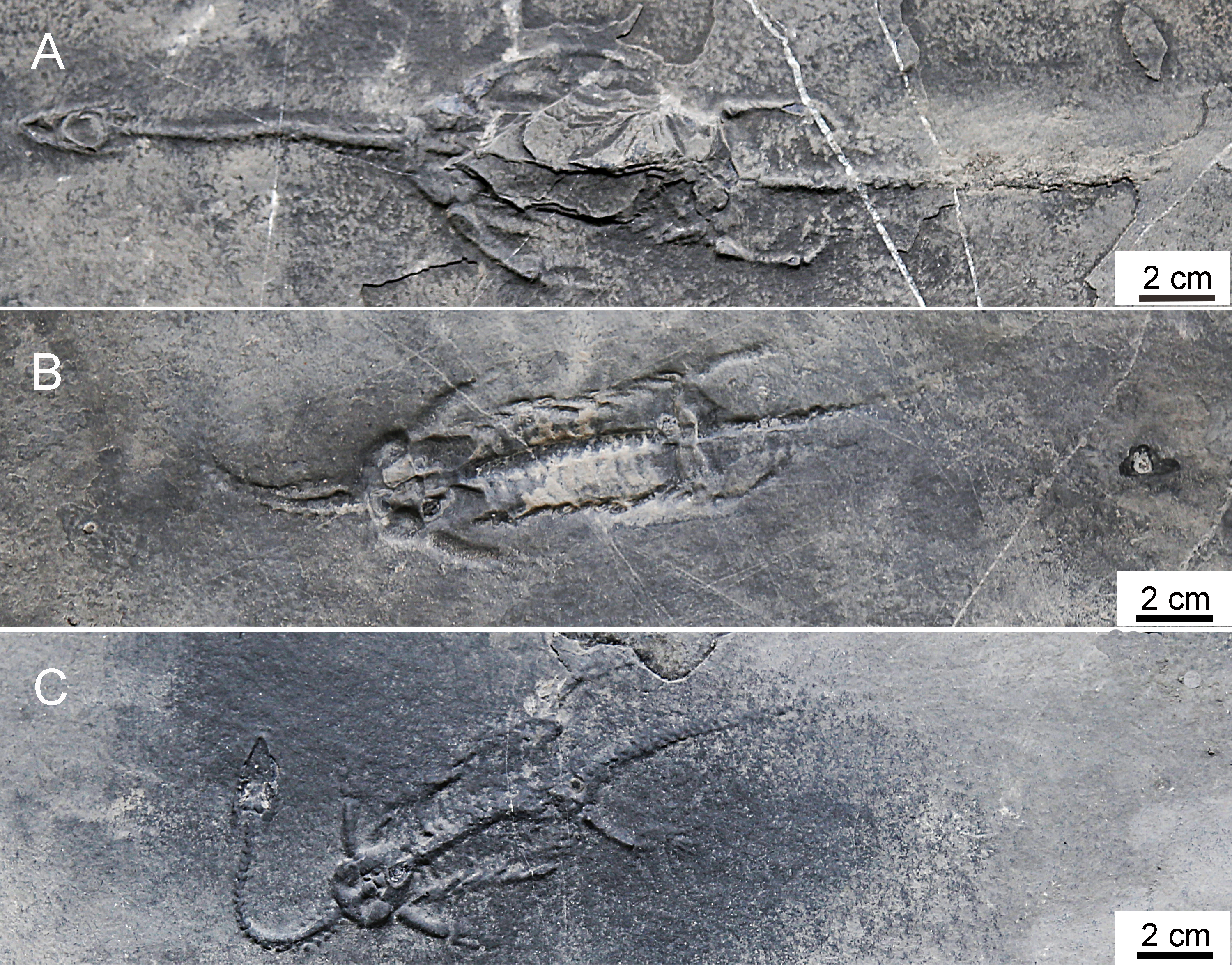

Supplement: Figure S1 — (A) CUGW VH007; (B) CUGW VH009; (C) CUGW VH017; all in ventral view. [file peerj-13-19012-s002.png]

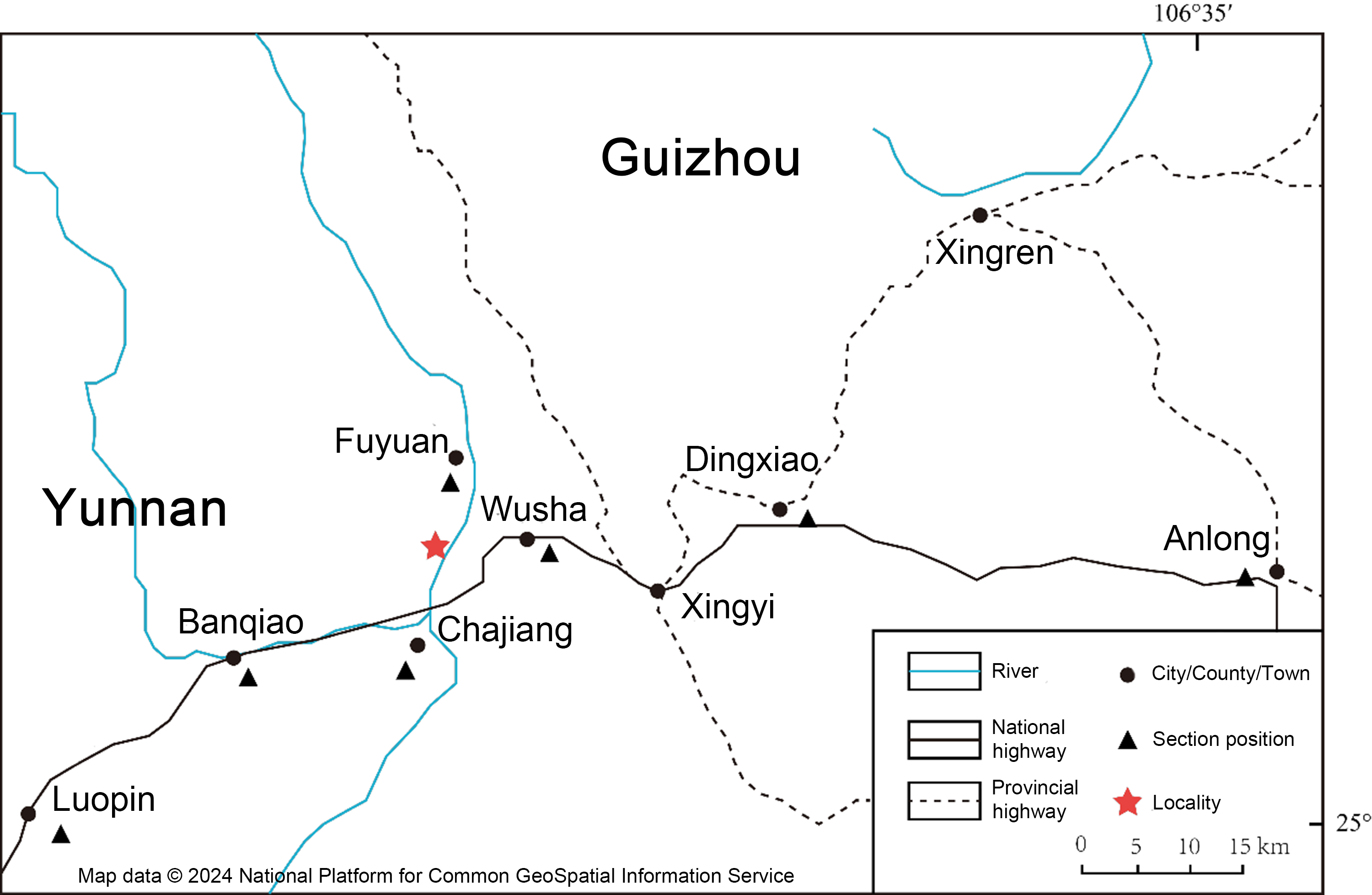

Supplement: Figure S2 [file peerj-13-19012-s003.png]
